# *Abelmoschus esculentus* subfractions attenuate Aβ and tau by regulating DPP-4 and insulin resistance signals

**DOI:** 10.1186/s12906-020-03163-4

**Published:** 2020-12-02

**Authors:** Chien-Ning Huang, Chau-Jong Wang, Chih-Li Lin, Hsin-Hua Li, An-Ting Yen, Chiung-Huei Peng

**Affiliations:** 1 Department of Internal Medicine, Chung-Shan Medical University Hospital, Taichung, Taiwan; 2 Institute of Medicine, Chung-Shan Medical University, Taichung, Taiwan; 3 Institute of Biochemistry, Microbiology and Immunology, Chung-Shan Medical University, Taichung, Taiwan; 4 Division of Basic Medical Science, Hungkuang University, Taichung City, Taiwan

**Keywords:** *Abelmoschus esculentus*, Beta amyloid, Insulin resistance, Dipeptidyl peptidase-4

## Abstract

**Background:**

Insulin resistance could be associated with the development of Alzheimer disease (AD). The neuropathological hallmarks of AD are beta amyloid (Aβ) produced from sequential cleavage initiated by β-secretase and degraded by insulin degradation enzyme (IDE), as well as hyperphosphorylation of tau (p-tau). Insulin action involves the cascades of insulin receptor substrates (IRS) and phosphatidylinositol 3-kinase (PI3K), while phosphorylation of IRS-1 at ser307 (p-^ser307^IRS-1) hinders the response. Our previous report suggested dipeptidyl peptidase-4 (DPP-4) is crucial to insulin resistance, and the subfractions of *Abelmoschus esculentus* (AE), F1 and F2, attenuate the signaling. Here we aim to investigate whether AE works to reduce Aβ generation via regulating DPP4 and insulin resistance.

**Methods:**

The subfractions F1 and F2 were prepared according to a succession of procedures. F1 was composed by quercetin glycosides and triterpene ester, and F2 contained a large amount of polysaccharides. The in vitro insulin resistance model was established by SK-N-MC cell line treated with palmitate. MTT was used to define the dose range, and thereby Western blot, ELISA, and the activity assay were used to detect the putative markers. One-way ANOVA was performed for the statistical analysis.

**Results:**

Treatment of palmitate induced the level of p-^ser307^IRS-1. Both F1 and F2 effectively decrease p-^ser307^IRS-1, and recover the expression of p-PI3K. However, the expression of total IRS plunged with 25 μg/mL of F1, while descended steadily with 5 μg/mL of F2. As palmitate increased the levels of Aβ40 and Aβ42, both AE subfractions were effective to reduce Aβ generation of and β-secretase activity, but IDE was not altered in any treatment conditions. The expression of DPP4 was also accompanied with insulin resistance signals. Inhibition of DPP4 attenuated the activity of β-secretase and production of Aβ. Moreover, the present data revealed that both AE subfractions significantly decrease the level of p-Tau.

**Conclusions:**

In conclusion, we demonstrated that AE would be a potential adjuvant to prevent insulin resistance and the associated pathogenesis of AD, and F2 seems more feasible to be developed.

## Background

Insulin resistance is related with obesity and metabolic syndrome. The worldwide increasing prevalence of diabetes, especially the type 2 characterized by insulin resistance and hyperinsulinemia, burdens the public health. Likewise, the incidence of Alzheimer disease (AD) is raised at alarming rate in aging society of many developed countries [1]. It is noteworthy that diabetic individuals showed higher risk to develop AD [2, 3], and the clinical observation revealed the association is independent of vascular factor [4].

The neuropathological hallmarks of AD are intercellular beta amyloid (Aβ) accumulation and intracellular neurofibrillary tangling. Aβ is a 38- to 43-amino acid peptide which is produced by sequential cleavage of amyloid precursor protein (APP) with β and γ-secretase, respectively. Insulin degradation enzyme (IDE) is one of the major enzymes degrading Aβ. An imbalance between production and clearance of Aβ forms senile plaque, an early and often initiating factor of AD. The neurofibrillary tangling is composed of hyperphosphorylated tau (p-tau), a group of proteins assembly to stabilize microtubules. Both Aβ and p-tau spread in a progressive manner with characteristic “regional specificity”, eventually leading to neuron loss and memory impairment [5].

In addition to exist in peripheral organs, insulin receptors also appear in central nervous system, especially hippocampus and cerebral cortex which play the pivotal role in learning and memory [6]. After binding to its receptor, the insulin action involves a series of cascades by eliciting tyrosine phosphorylation of insulin receptor substrates (IRS), leading to the activation of phosphatidylinositol 3-kinase (PI3K). An increase of phosphorylation of IRS-1 at the residue ser307 (p-^ser307^IRS-1) hinders the insulin response and glucose utilization, thus viewed as the insulin resistance marker [7]. Moreover, as dipeptidyl peptidase-4 (DPP-4) inhibitors have emerged as a useful tool in treating type 2 diabetes, our previous reports showed that DPP-4 activity is crucial to the downstream insulin resistance signals [8].

In literature, it has been shown the close relationship between insulin and Aβ metabolism. Insulin resistance accelerated the assembly of Aβ by inducing GM1 ganglioside clustering in the presynaptic membranes [9]. Insulin raised the extracellular level of Aβ by increasing its release [10], or modulating the secretase activity [11]. IDE could be competitively inhibited by insulin, resulting in decreased degradation of Aβ [12, 13]. It was suggested IDE acts as the junction point of type 2 diabetes and AD [14]. Induced by excessive sucrose intake, insulin resistance increased cerebral Aβ peptide levels and exacerbated learning impairment in AD transgenic mice [15]. In AD-like mouse model, combination of insulin with GLP1 agonist reduced the expression of effector genes involved in insulin receptor signaling. Cortical Aβ levels were also decreased by 15–30%, accompanied with better learning [16]. These revealed that there is the close relation between AD and insulin resistance.

*Abelmoschus esculentus* (AE) fruit is consumed as vegetable and famous in folklore medicine because of its anti-diabetic effect [17, 18], whereas the substantial mucilage makes it difficult to test the active components. In our previous studies, we isolated several subfractions from AE using a series of successive extraction steps, and found that F1 (the subfraction rich in quercetin glucosides and pentacyclic triterpene ester) and F2 (the subfraction containing large amounts of carbohydrates and polysaccharides) were particularly effective in suppressing DPP-4 signaling [19–21]. Recently, we reported that AE is potential to prevent Aβ-induced neuron damage by regulating DPP-4 and the insulin resistance cascades [22].

In the present study, we aimed to investigate whether AE could be effective to attenuate Aβ generation and metabolism, via regulating DPP4 and insulin resistance cascades.

## Methods

### Preparation of AE subfractions and chemical analysis

The fruit of AE was purchased from Chiayi, Taiwan. The subfractions F1 and F2 were prepared according to a succession of procedures [19]. F1, the alcohol-extracted fraction of AE, has been analyzed with LC-MS/MS. At least 10 compounds were found in F1, including quercetin glucosides (4.901 mg/g DW) and pentacyclic triterpene ester (4.301 mg/g DW) (The content was estimated in comparison with the standard). F2 contains a large amount of polysaccharides. With gel filtration chromatography (GPC) analysis, the mean molecular weight of F2 was estimated to be 671 kDa. The monosaccharide analysis and uronic determination revealed that F2 is rich in uronic acid (23.14%), galactose (18.92%), glucose (18.26%) and myo-inositol (14.21%); rhamnose, fucose and glucosamine were also found to be quite abundant [19].

### Cell culture

The cell line SK-N-MC was obtained from American Type Culture Collection (ATCC). Cells were cultured in Minimum Essential Medium (MEM), with 2 mM L-glutamine and penicillin-streptomycin, and 10% fetal bovine serum (FBS), and grown in a humidified incubator at 37 °C, 5% CO2. The medium was changed every 2 or 3 days. When the cells reached approximately 80% confluency, the subcultures were undergone. The phase contrast microscope with photo-system was used for image observation.

### MTT

Cells were seeded on the 24 well-plate at a density of 2 × 10^5^ cells/mL. After attachment, cells were incubated with palmitate with or without AE subfractions at various concentrations for 24 h. The medium was changed and cells were incubated with 0.5 mg/mL 3-(4, 5-dimethyl-2-thiazol)-2, 5-diphenyltetrazolium bromide (MTT, CAS 298–93-1) for 2 h. The amount of viable cells was directly proportional to the production of formazan. Following the dissolution with isopropanol (1 mL/well) and centrifugation (at 12000 rpm), each sample was added into a 96 well-plate (200 μL per well), and the absorbance was measured on spectrophotometer at 563 nm (Hitachi, U-3210).

### Western blot

Cells were harvested with lysis buffer containing 50 mM Tris HCl (pH 6.8), 10% glycerol, 2% SDS, and 5% mercaptoethanol, and then lysed by sonication. Cell lysate was centrifuged at 9300 *g* for 20 min at 4 °C, and then the supernatant was collected as protein samples. After quantification with Bradford assay, equal amount of protein sample (50 μg) was subjected to 10% SDS-polyacrylamide gel electrophoresis, and transferred to nitrocellulose membranes (Millipore, Bedford, MA, USA). The membranes were blocked with 5% nonfat milk powder with 0.1% Tween-20 in TBS, and incubated with the primary antibody overnight at 4 °C against the following targets: pPI3K (1:200), PI3K (1:200), p-^ser307^IRS-1 (1:1000), IRS-1(1:200), IDE (1:5000), and p-Tau (1:1000). The antibody of PI3K and IRS-1 were from Santa Cruz (Sc-374,534 and Sc-7200, respectively), and p-PI3K was from Bioss (Bs-3332R). Antibodies of p-^ser307^IRS-1 was from Cell Signaling Technology (2381S), p-Tau was from Merck Millipore (MAB5450), and IDE was from Thermo (PA5–29350). After this, the membranes were washed three times with 0.1% Tween-20 in TBS, and incubated with the secondary antibody (1:5000) conjugated to horseradish peroxidase (GE Healthcare, Little Chalfont, Buckinghamshire, UK). The band detection was thereafter fulfilled by enhanced chemiluminescence with ECL western blotting detection reagents and FUJFILM Las-3000 (Tokyo, Japan). Protein quantity was determined by densitometry using FUJFILM-Multi Gauge V2.2 software.

### DPP-4 activity

Cells were seeded on 6 cm-dish at a density of 1 × 10^6^. After treated with various conditions, cells of each well were lysed with 100 μL of NP-40 lysis buffer (containing 10 mM HEPES (pH 7.5), 142.5 mM KCl, 5 mM MgCl2, 1 mM EGTA, and 0.2% NP-40), and then centrifuged at 9300 *g* for 20 min at 4 °C. The supernatants were collected, and the protein concentrations were determined with Bradford assay. DPP4 activity was measured using DPP4/ CD26 assay kit for biological samples (Enzo Life Sciences). Briefly, H-Gly-Pro-pNA, a chromogenic substrate of DPP-4, was hydrolysed into dipeptide Gly-Pro and 4-nitroaniline, whose appearance rate was measured spectrophotometrically at 405 nm. The activity was normalized with protein concentration, and then leveled in proportion to the control. For analyzing the putative role of DPP-4, linagliptin was used to inhibit the activity of DPP-4.

### Aβ generation

Cells were treated with different conditions, with or without 50 μM of palmitate added with F1 (1, 5, and 25 μg/mL, respectively) or F2 (1, 2.5, and 5 μg/mL, respectively), and the medium was collected. Each sample was analyzed with Aβ42 and Aβ40 ELISA Kit, which are the products of Invitrogen, encoded as #KHB3441 and #KHB3481, respectively,

### β-Secretase activity

Beta-secretase Activity kit (fluorometric), was the product of Abcam (#ab65357). The protocol uses a secretase-specific peptide conjugated with two reporter molecules (EDANS and DABCYL). Cleavage of the peptide by beta secretase physically separates the two molecules, allowing for the release of fluorescent signals.

### Statistical analysis

The statistical software SPSS v.12.0 was used to analyze the data. One-way ANOVA was performed (*p* < 0.05), while Bonferroni’s multiple comparison was used for post-test.

## Results

### Concentrations applied were tested for the safety range

**Fig. 1:**
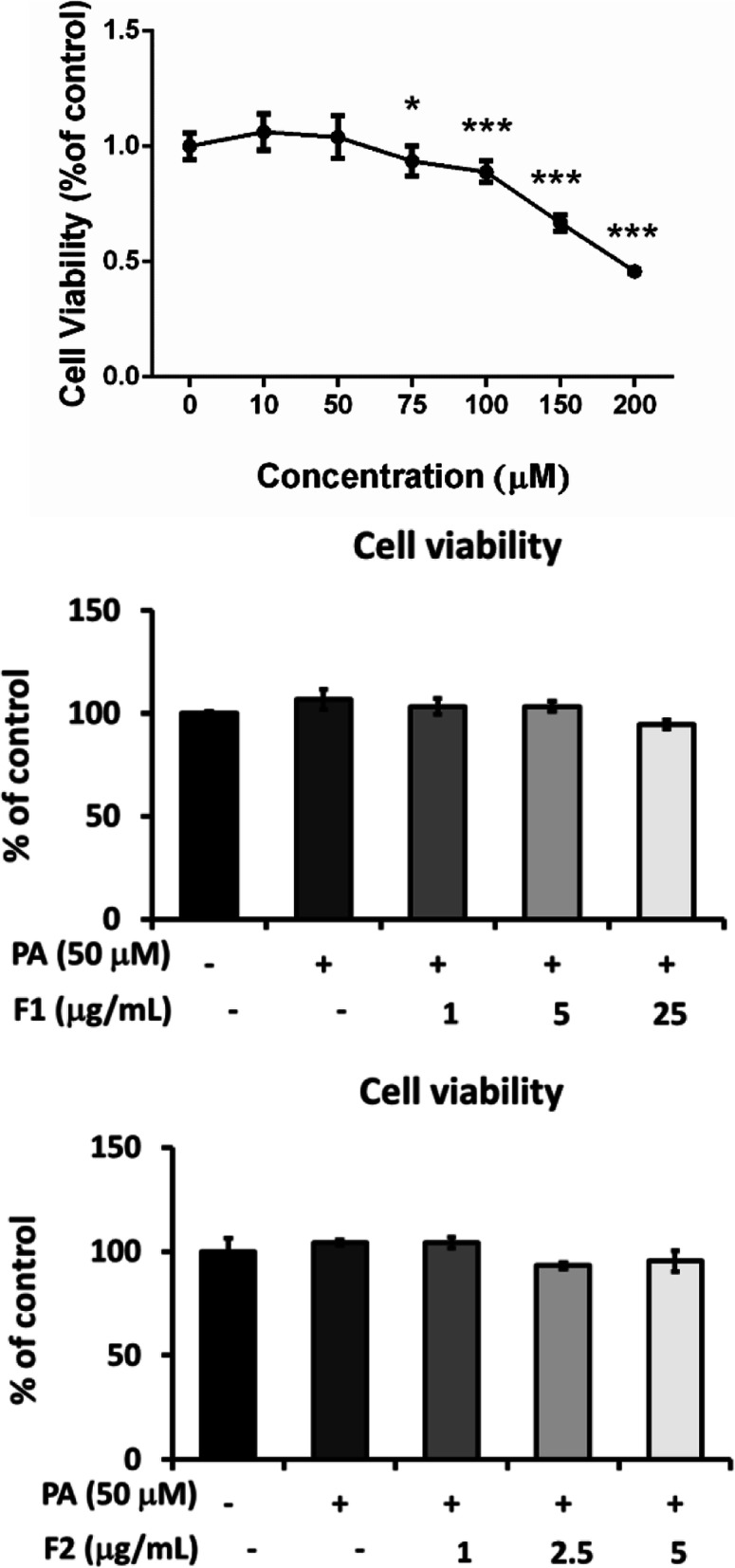
Cytotoxicity of palmitate and AE subfractions. SK-N-MC cells were incubated for 24 h with or without different concentrations of palmitate. The cell viability was analyzed with MTT and calculated as a percentage compared with that of the control group. 50 μM of palmitate was determined to be used in the following experiment, treated with different concentrations of F1 and F2. All the data were presented as means ± SD (*n* = 3), and statistically analyzed with ANOVA. *p < 0.05, **p < 0.01, ***p < 0.001, compared with the control

In the present study, palmitate was used to induce insulin resistance but not cell death. At beginning of the experiment, we used MTT to test the operating dose of palmitate. Figure 1 showed that palmitate reduced the cell viability dose-dependently above 50 μM. As for AE, F1 and F2 had been tested to the cell line SK-N-MC in our previous work, and the maximum dose were defined as 25 μg/mL and 5 μg/mL, respectively [22]. Treatment of 50 μM of palmitate with different doses of F1 and F2 revealed that it would not harm the cell. Hence these dose ranges were chosen and applied in the following manipulation.

### AE attenuate insulin resistance signals induced by palmitate

**Fig. 2:**
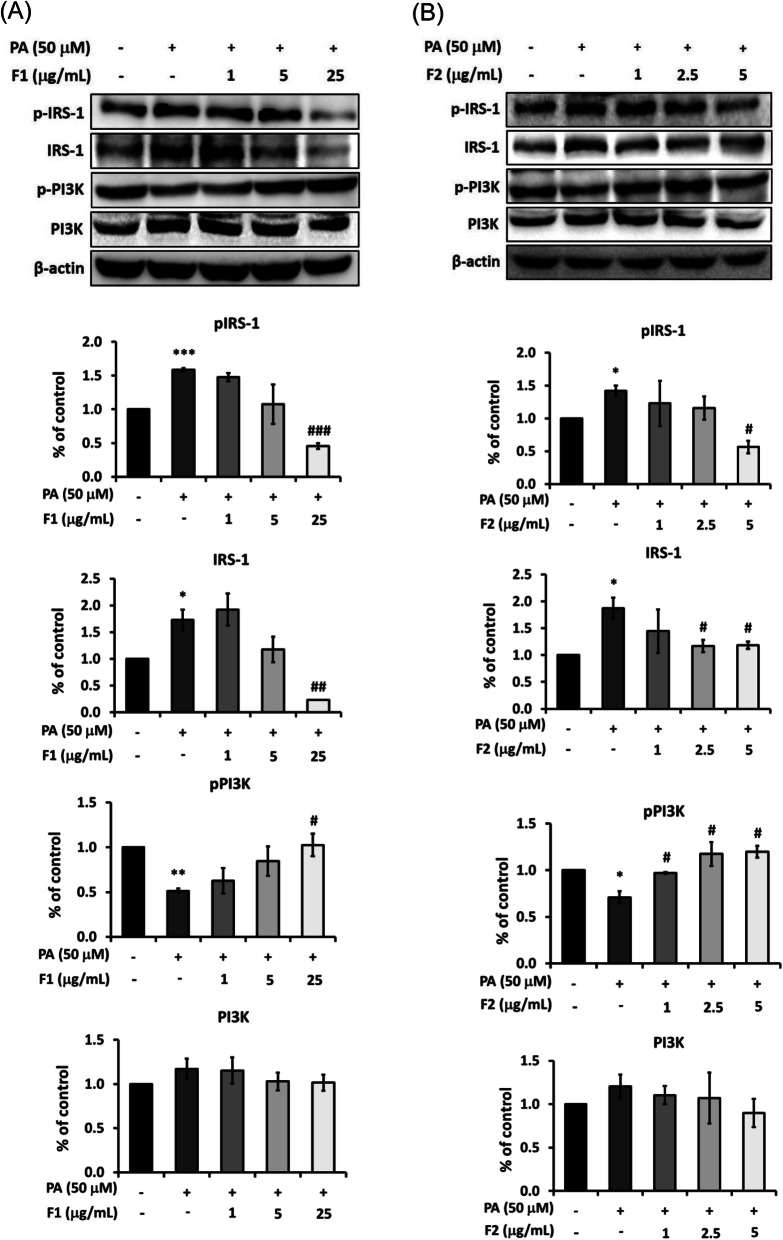
Effect of AE on attenuating insulin resistance signals. SK-N-MC cells were incubated for 24 h with or without 50 μM of palmitate, and with different concentrations of F1 (**a**) and F2 (**b**). The expressions of p-^ser307^IRS-1, IRS-1, p-PI3K and PI3K were analyzed by Western blotting. Data were presented as means ± SD (*n* = 3), and analysed with ANOVA. *p < 0.05, **p < 0.01, ***p < 0.001, compared with the control. #*p* < 0.05, #*p* < 0.01, #*p* < 0.001 compared with the palmitate-treated only

Figure 2a showed that palmitate increased the phosphorylation of p-^ser307^IRS-1 about 1.5 folds. Treatment with F1, at 25 μg/mL, significantly decreased p-^ser307^IRS-1 to the level even below the control. The expression of total IRS was also increased by palmitate, and extremely lowered by 25 μg/mL of F1. In contrast, the expression of p-PI3K was half-reduced by palmitate and returned by F1, while the expression of PI3K was altered neither by palmitate nor F1.

F2 had the similar ability to counterwork palmitate (Fig. 2b). At 5 μg/mL, F2 effectively attenuated the level of p-^ser307^IRS-1 and IRS. It is noteworthy that even at a low dose of 1 μg/mL, F2 significantly recovered the phosphorylation of p-PI3K, and 2.5 and 5 μg/mL of F2 increased p-PI3K to the level more than the control.

**Fig. 3:**
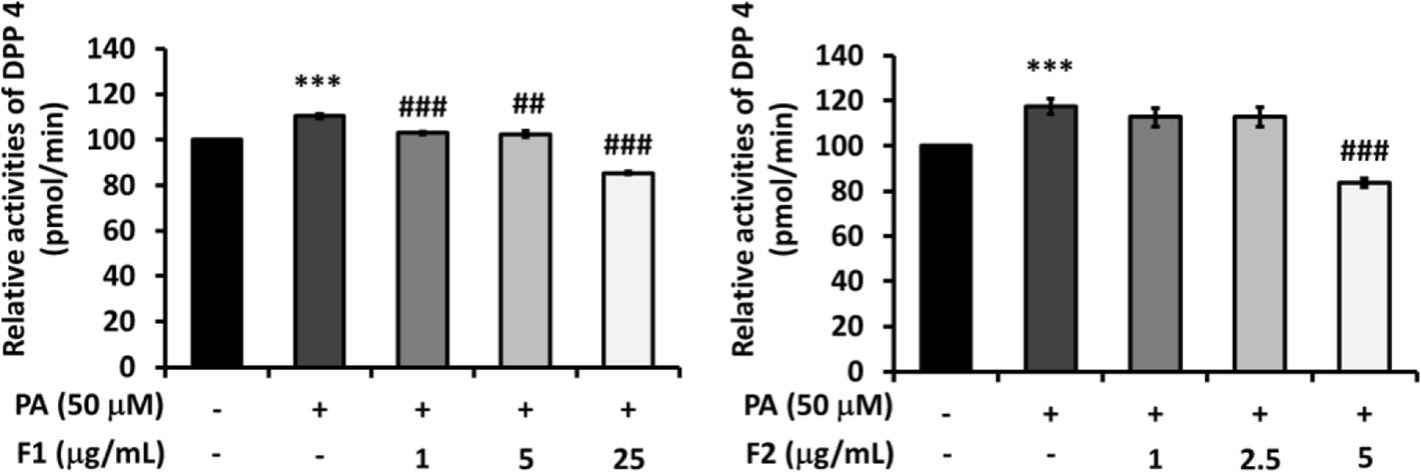
Effect of AE on attenuating DPP-4 activity. SK-N-MC cells were incubated for 24 h with or without palmitate, and with different concentrations of F1 and F2, thereby analyzed with DPP-4 activity. The activity of DPP-4 was calculated as a percentage compared with that of the control group. Data were presented as means ± SD (*n* = 3), and analysed with ANOVA. *p < 0.05, **p < 0.01, ***p < 0.001, compared with the control. #*p* < 0.05, #*p* < 0.01, #*p* < 0.001 compared with the palmitate-treated only

Figure 3 showed that palmitate significantly induced the activation of DPP4, while both F1 and F2 were quite effective to down-regulate the activation.

Compared to our previous report, these data of Figs. 2 and 3 revealed that palmitate triggers insulin resistance signals by elevating DPP4 and p-^ser307^IRS-1, and reducing p-PI3K. AE subfractions attenuate DPP4 activation and the cascades of insulin resistance.

**Fig. 4:**
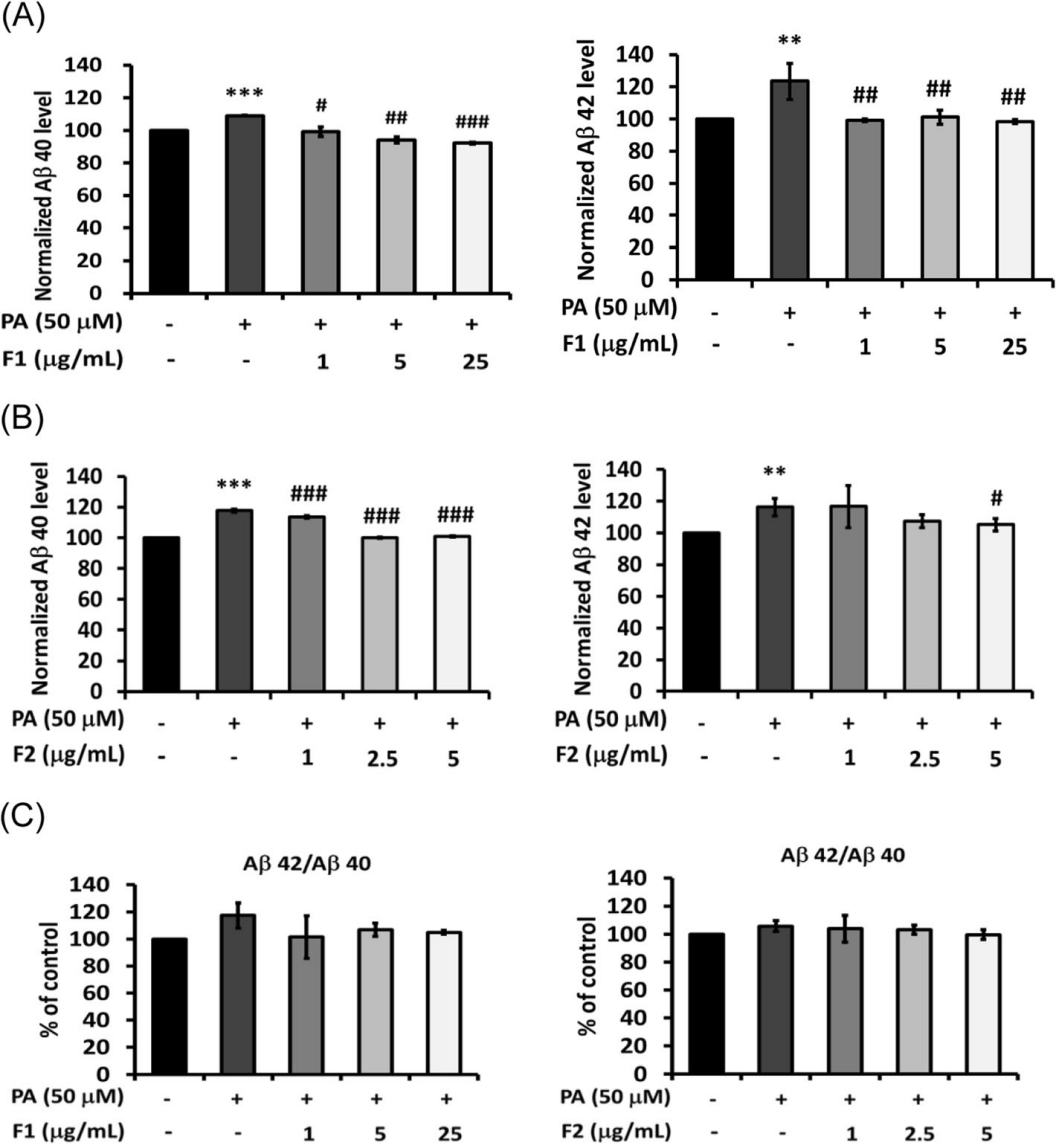
Effect of AE on attenuating Aβ generation. SK-N-MC cells were incubated for 24 h with or without palmitate, and with different concentrations of F1 (**a**) and F2 (**b**), thereby analyzed with Aβ generation. Aβ42/40 ratio is shown as (**c**). Data were presented as means ± SD (*n* = 3), and analysed with ANOVA. *p < 0.05, **p < 0.01, ***p < 0.001, compared with the control. #*p* < 0.05, #*p* < 0.01, #*p* < 0.001 compared with the palmitate-treated only

### AE attenuate Aβ generation induced by palmitate

**Fig. 5:**
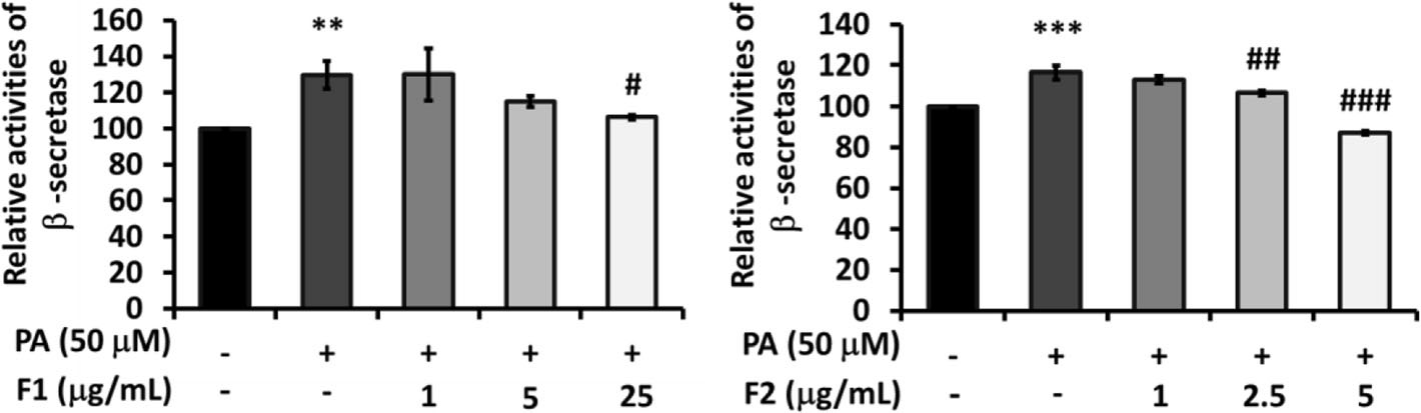
Effect of AE on attenuating β-secretase. SK-N-MC cells were incubated for 24 h with or without palmitate, and with different concentrations of F1 and F2, thereby analyzed with β-secretase activity. Data were presented as means ± SD (*n* = 3), and analysed with ANOVA. *p < 0.05, **p < 0.01, ***p < 0.001, compared with the control. #*p* < 0.05, #*p* < 0.01, #*p* < 0.001 compared with the palmitate-treated only

**Fig. 6:**
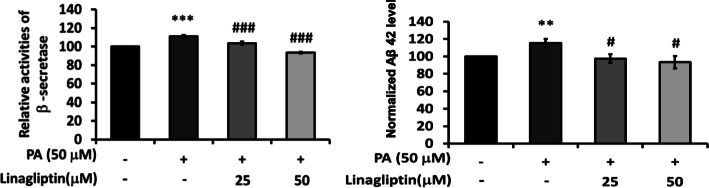
DPP involved in the expressions of Aβ and β-secretase. SK-N-MC cells were incubated for 24 h with or without palmitate, and with different concentrations of linagliptin, thereby analyzed with Aβ generation and β-secretase activity. Data are presented as means ± SD (*n* = 3) and analysed with ANOVA. *p < 0.05, **p < 0.01, ***p < 0.001, compared with the control. #*p* < 0.05, #*p* < 0.01, #*p* < 0.001 compared with the palmitate-treated only

As palmitate increased the generation of Aβ40 and Aβ42, F1 and F2 were effective to reduce the levels of Aβ. No significant change was observed for Aβ42/40 (Fig. 4). Palmitate enhanced the activity of β-secretase about 1.2 folds, while 25 μg/mL of F1 could significantly inhibit β-secretase. F2, at 5 μg/mL, lowered β-secretase activity to even below the control (Fig. 5). To investigate whether DPP4 could be involved in the metabolism of Aβ40 and Aβ42, linagliptin (one of the DPP4 inhibitors) was added with palmitate. It was shown that the inhibition of DPP4 was quite valid to attenuate β-secretase and Aβ generation. These results imply that DPP4 and its signal cascades could be critically involved in the metabolism of Aβ40 and Aβ42 (Fig. 6).

### AE attenuate the expression of tau

**Fig. 7:**
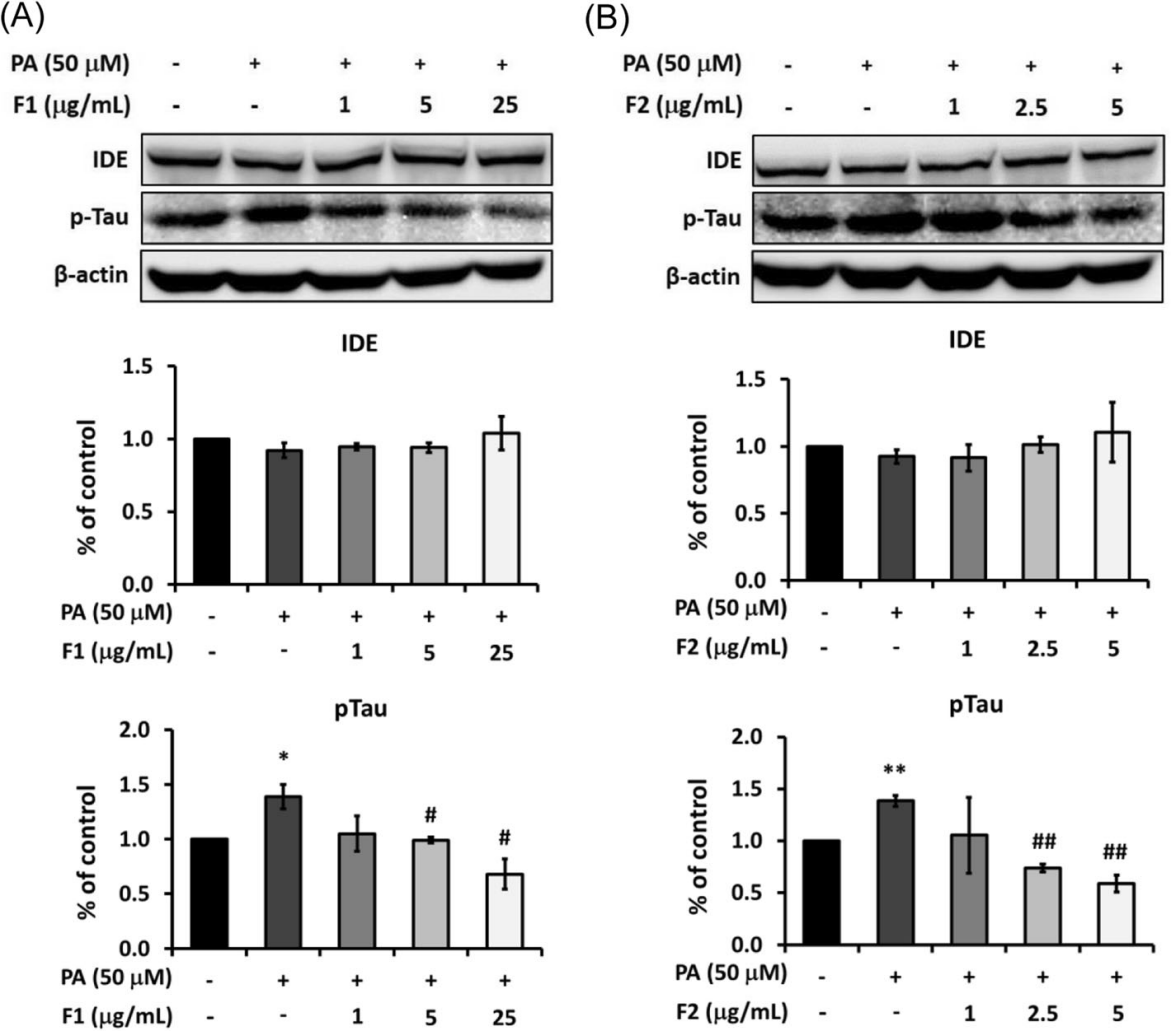
Effect of AE on attenuating p-Tau. SK-N-MC cells were incubated for 24 h with or without 50 μM of palmitate, and with different concentrations of F1 (**a**) and F2 (**b**). The expressions of IDE and p-Tau were analyzed by Western blotting. Data are presented as means ± SD (*n* = 3) and analysed with ANOVA. *p < 0.05, **p < 0.01, ***p < 0.001, compared with the control. #*p* < 0.05, #*p* < 0.01, #*p* < 0.001 compared with the palmitate-treated only

The levels of Aβ40 and Aβ42 could be influenced by the process of Aβ degradation. Therefore, in addition to Aβ generation, we observed the expression of IDE. Figure 7 showed that IDE was not altered by 50 μM of palmitate. As well, treatment of AE did not affect any expression of IDE.

On the other hand, Tau is another hallmark of AD apart from Aβ. It was shown that palmitate induced the phosphorylation of Tau near 1.5 folds. F1, at 5 and 25 μg/mL, significantly reduced the expressions of Tau (Fig. 7a). F2, at 2.5 μg/mL, was enough to lower p-Tau to the level below the control. At 5 μg/mL, F2 even reduced 40% of p-Tau, compared with the control (Fig. 7b).

## Discussion

In the present study, we demonstrated AE attenuate palmitate-induced DPP-4 activation and insulin resistance cascades. Both F1 and F2 effectively decrease the level of p-^ser307^IRS-1, and recover the expression of p-PI3K. As palmitate enhances the level of Aβ_,_ AE significantly suppress Aβ generation and β-secretase activation, while IDE was not altered. The inhibition of DPP4 is quite valid to attenuate β-secretase activity and the production of Aβ, implying DPP4 and its signal cascades could be critically involved in Aβ metabolism. Moreover, both F1 and F2 significantly decrease the level of Tau, another hallmark of Alzheimer besides Aβ.

Aβ, especially the insoluble 40- to 42-amino acid peptide named Aβ40 and Aβ42 respectively, is the principal component of the cerebral plaques found in the brains with Alzeheimer’s disease (AD). Aβ is formed by the cleavage of APP. Three proteases α-, β-, and γ-secretases cleave APP, and thus be implicated in the etiology of AD. Alpha-secretase exhibits characteristics of certain membrane-tethered metalloproteases. Beta-Secretase is a membrane-anchored protein with clear homology to soluble aspartyl proteases. Gamma-Secretase is an oligomeric complex including presenilins, the pupative catalytic component of this protease [23]. It is well known that two principal metabolic pathways are involved in the processing of APP. The “β-secretase” pathway mainly generates Aβ40/42 (4-kD) by sequential cleavage with β- and γ-secretase, and the “α-secretase” pathway generates a smaller peptide P3 (3-kD) cleaved with α- and γ-secretase [24]. The present study focused on Aβ metabolism and “β-secretase” pathway. Since the activity of γ-secretase could be involved in another pathway, we evaluated merely β-secretase but not γ-secretase. Meanwhile, the level of Aβ could be influenced by its degradation. However, our data showed that the expression of IDE was not altered by palmitate or AE. It can be implied that the dose range applied or the signal cascades are nothing to do with IDE.

Our data showed AE significantly attenuate Aβ40 and Aβ42 induced by palmitate. In fact, Aβ42 aggregates to provide a nidus for the subsequent aggregation of Aβ40, leading to the formation of innumerable neuritic plaques [25]. A spectroscopic analysis revealed that dimeric Aβ, derived from mutant APP with a single cysteine in the ectodomain juxtamembrane position, display much more pronounced structural transition than its corresponding monomers. In vivo studies also revealed that two forms of Aβ homodimers (Aβ40 and Aβ42) exist, and the dimerization increased beta-sheet content. As reported, the dimeric forms with C-terminal residues Ile-41 and Ala-42 further increased the beta-sheet content by roughly one-third [26]. It was suggested that the neurotoxic core of Aβ is neurotoxic only when it forms beta-sheet and aggregates [27].

In our previous report, we had demonstrated DPP-4 mediates the insulin resistance signals, leading to the pathological phenomenons associated with diabetes and its complications. Accompanied with increasing p-^ser307^IRS-1 and decreasing p-PI3K, palmitate did hinder glucose uptake in the presence of insulin. Treatment with the polyphenol extracts of *Hibiscus sabdariffa* (HPE) and DPP-4 inhibitor linagliptin completely recovered insulin sensitivity and palmitate-induced signal cascades, thus decreased AT-1-mediated tubular epithelial to mesenchymal transition [8]. In β cells, palmitate induced the signals of DPP-4, p-PI3K and p-AMPK, and AE and linagliptin attenuated the signaling cascades. Conversely, palmitate downregulated mTOR, while both F1 and F2 significantly restored the level of mTOR [21]. Actually, in the clinical trial, DPP-4 inhibitor lowered the albuminuria in type 2 diabetic patients, and this was independent of the level of HbA1C [28]. The present investigation showed that linagliptin attenuate palmitate-induced β-secreatse activity and Aβ generation, implying that DPP-4 could take part in regulating p-^ser307^IRS-1 and p-PI3K, and mediate the development of AD. Noticeably, our recent report indicated that AE was potential to prevent Aβ-induced neuron apoptosis by reducing DPP-4 and p-^ser307^IRS-1, and enhancing p-PI3K and p-AMPK. Treatment of linagliptin attenuated the neuron apoptosis induced by Aβ [22]. These results suggest that reciprocal regulations could exist between Aβ metabolism and insulin resistance.

The hyperphosphorylation of Tau is another hallmark of AD. In high fat diet-induced obesity and insulin resistance, the supplementation with *Myrciaria jaboticaba* berry increased insulin sensitivity, thus prevented tau phosphorylation and improved learning/memory performance [29]. The present data showed that AE attenuate palmitate-induced p-Tau, In comparison with our previous report which revealed Aβ increased p-Tau, AE is supposed to attenuate the microtubular change via improving insulin resistance and Aβ generation [22].

F1 and F2 were suggested useful to improve diabetes, insulin resistance, and the associated complications. Quercetin, the component rich in F1, improved cognitive function by inhibiting neurofibrillary tangles mediated by phosphorylated Tau [30]. F2, the most effective AE subfraction in attenuating hyperglycemia and insulin resistance [31], is composed of polysaccharide and sugar derivatives including myoinositol. It was reported intragastric administration of *Codonopsis pilosula* polysaccharide rescued p-Tau overexpression-induced cognitive defects [32], and the myoinositol was identified as a molecule inhibiting β-secretase [33]. Hence the effect of F2 could be at least partly attributed to its polysaccharide and myoinositol. Furthermore, besides the alteration of p-^ser307^IRS-1, the protein level of IRS-1 displayed a similar change (Fig. 2). This phenomenon may be attributed to the compensation of IRS. In non-diabetic rats, myocardial infarction induced by artery ligation leads to a partial impairment of insulin response. The similar defect might occur in human patients, resulting in compensatory increase of IRS expression [34]. However, the expression of total IRS plunged with 25 μg/mL of F1, but descended steadily with 5 μg/mL of F2. It cannot be ruled out that the phosphorylation change of F1 was caused by destabilizing of protein.

## Conclusions

Therefore, despite both subfracttions of AE attenuate palmitate-induced insulin resistance and hallmark expression of AD, F2 seems more feasible to be developed. In conclusion, we demonstrated that AE would be a potential adjuvant to prevent insulin resistance and the associated pathogenesis of AD.

## Data Availability

All data/material is available on request from the corresponding author.

## Abbreviations

AD: Alzheimer disease
Aβ: Beta amyloid
APP: Amyloid precursor protein
IDE: Insulin degradation enzyme
p-tau: Hyperphosphorylation of tau
IRS: Insulin receptor substrates
PI3K: Phosphatidylinositol 3-kinase
p-ser307IRS-1: IRS-1 at ser307
DPP-4: Dipeptidyl peptidase-4
AE: *Abelmoschus esculentus*, F1 and F2
ATCC: American Type Culture Collection
MEM: Minimum Essential Medium
FBS: Fetal bovine serum
HPE: *Hibiscus sabdariffa*
